# Solvatochromic effect in absorption and emission spectra of star-shaped bipolar derivatives of 1,3,5-triazine and carbazole. A time-dependent density functional study

**DOI:** 10.1007/s00894-017-3234-y

**Published:** 2017-02-04

**Authors:** Gleb V. Baryshnikov, Sergey V. Bondarchuk, Valentina A. Minaeva, Hans Ågren, Boris F. Minaev

**Affiliations:** 1 Division of Theoretical Chemistry and Biology, School of Biotechnology, KTH Royal Institute of Technology, 10691 Stockholm, Sweden; 2 Department of Chemistry and Nanomaterials Science, Bogdan Khmelnitsky Cherkasy National University, blvd. Shevchenko 81, 18031 Cherkasy, Ukraine

**Keywords:** Star-shaped compounds, OLEDs, TDDFT, Solvatochromic effect, Dipole moment

## Abstract

A series of three star-shaped compounds containing both donor (carbazole) and acceptor (2,4,6-triphenyl-1,3,5-triazine) moieties linked through various linking bridges was studied theoretically at the linear response TD-DFT level of theory to describe their absorption and fluorescence spectra. The concept of a localized charge-transfer excited state has been applied successfully to explain the observed strong solvatochromic effect in the emission spectra of the studied molecules, which can be utilized for the fabrication of color tunable solution-processable OLEDs. The concept is in particularly applicable to donor–acceptor species with a *C*_3_ symmetry point group where the static dipole moment changes dramatically upon electronic excitation. An important peculiarity of the studied molecules is that they are characterized by non-zero values of the HOMO and LUMO orbitals in the same common part of molecular space that provides a large electric dipole transition moment for both light absorption and emission.

**Electronic supplementary material:**

The online version of this article (doi:10.1007/s00894-017-3234-y) contains supplementary material, which is available to authorized users.

**Graphical abstract:**
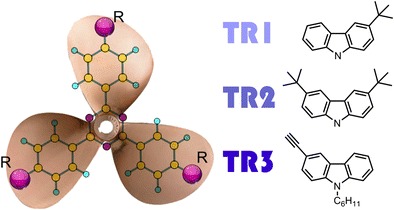
Star-shaped *C*_3_ symmetry point group derivatives for color tunable OLEDs

## Introduction

In recent years, star-shaped organic materials have attracted a great deal of attention due to their promising applications in organic light-emitting devices (OLEDs) [1–6]. One of the main challenges in this field is the realization of ambipolar transporting properties within single-type molecules, together with the strong photoluminescence that allows improvement of the luminance characteristics of OLEDs [5]. Another important feature of star-shaped organic luminophores is that they tend to form exciplexes with a wide range of organic materials, which is very useful for the emission-color tuning ability of OLEDs [7–9].

The general strategy to create ambipolar star-shaped emitters is to combine donor (D) and acceptor (A) fragments within the same molecule [5, 6], which (1) facilitates injection and transport properties of both charge-carriers-holes and electrons, and (2) activates intermolecular charge-transfer (CT) excited states in the photoluminescence spectra. Such CT states usually are the lowest-lying excited states of the star-shaped molecules, i.e., they are responsible for the fluorescence process. Moreover, the nonzero values of the HOMO and LUMO wave-functions in the same common part of the molecular space is a very important property of efficient star-shaped emitters [10, 11], providing a large electric dipole transition moment for both light absorption and emission [12].

It is well-known that D-A molecules usually demonstrate solvent-dependent behavior in their absorption spectra due to the high polarization of the ground-state molecular structure [12–17]. Strong positive solvatochromism in absorption spectra is observed frequently for molecules with a ππ* nature of band-productive electronic state. The absolute value of the red shift depends, usually linearly, on the solvent polarity (the higher the solvent polarity, the stronger the red shift) [6]. This is because the more polar solvent species polarizes molecules with a higher static dipole moment more strongly (particularly for D-A systems). But, in the case of symmetrical star-shaped molecules (3D-A, for example), the static dipole moment of the whole system is equal to zero because of the *C*_3_ symmetry point group restriction. Therefore, these systems should not demonstrate strong solvatochromism in the absorption spectra. However, if the excited state of the star-shaped molecule corresponds to the local CT ππ*-state, the structure of the excited-state geometry should be significantly distorted and the dipole moment of the excited state should differ markedly from zero. This means that clear solvatochromic behavior should be observed in the emission (fluorescence) spectra of D-A star-shaped molecules with local CT excited states rather than in absorption spectra where the vertical excitation prevails. In this way we can vary the emission color of the star-shaped compound in various solvents using the same excitation energy, which is very useful for applications of *C*_3_ symmetry point group in photovoltaic cells, OLEDs and bioimaging technologies [6].

In the present work, we focused on the three recently synthesized star-shaped compounds containing both D (carbazole) and A (2,4,6-triphenyl-1,3,5-triazine) moieties connected through various linking bridges [6]. We describe the results of quantum-chemical calculations carried out in order to study the “structure–optical properties relationship” of these D-A materials. Such compounds demonstrate clear solvent-dependent fluorescence, but the solvent effect is less observable in the absorption spectra. We think that the selected star-shaped compounds are really good candidates to prove the theory of local CT excited states in organic fluorofores of the *C*_3_ symmetry point group.

## Computational details

The calculations presented in this paper were performed in terms of density functional theory (DFT) using the Gaussian09 suite of programs [18, 19]. Geometry optimizations were carried out by the hybrid exchange-correlation functional B3LYP [20, 21] with the Pople’s split-valence basis set (almost double-ζ in the valence shell, 6-31 G) and addition of polarization (d, p) functions [22, 23]. The optimized structures were checked for absence of imaginary frequencies in the vibrational spectra, and the geometries obtained were justified as global minima.

The UV-vis spectra were obtained in terms of time-dependent density functional theory (TD-DFT) [24]. For this purpose, we applied the conventional B3LYP scheme as well as the modified B3LYP functional with the changed contribution of the exact Hartree-Fock exchange (HFE) part, which was increased up to 30%. Furthermore, we used the PBE0 [25], mPW1PBE [26], CAM-B3LYP [27], BMK [28], ωB97XD [29], and M062X [30] functionals. Polar media simulations were performed in terms of the polarizable continuum model (PCM) using integral equation formalism (IEFPCM) [31]. To define cavities, the universal force field (UFF) radii were used. The overlap index and minimum radius of the spheres were specified as 0.8 and 0.5 Å, respectively. We calculated the energies and oscillator strength values for 15 vertical electronic transition using the same 6-31 G basis set as that used for geometry optimization. The fluorescence energies were computed, taking into account relaxation of the excited state geometry (TD-DFT optimization of the S_1_ state geometry), including the state-specific equilibrium solvation correction.

The electronic absorption spectra curves were fitted using the Gauss distribution function and a half-width of 3000 cm^−1^ with the SWizard 5.0 program package [32]. Molecular visualizations were performed with Chemcraft 1.6 [33].

## Results and discussion

### Effect of exact HFE on UV-vis spectra prediction

**Fig. 1:**
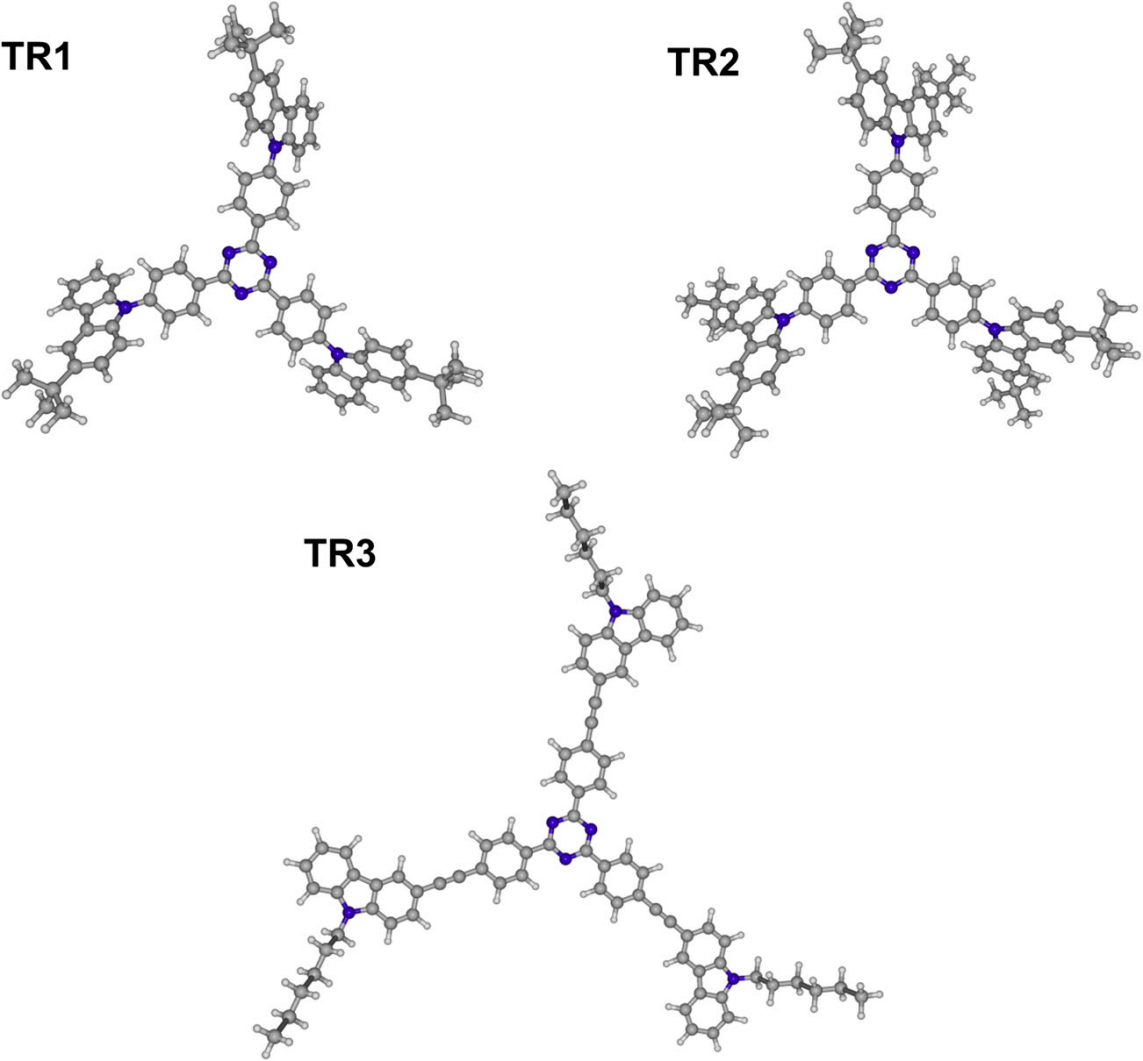
Structure of the species **TR1**–**TR3** optimized by the DFT(B3LYP)/6-31G(d,p) method in the *n*-hexane [polarizable continuum model (PCM) using integral equation formalism (IEFPCM)] solvent

The optimized structures of the studied propeller-shaped bipolar derivatives of 1,3,5-triazine and carbazole, namely, 2,4,6-tris(4-(3-tert-butyl-carbazol-9-yl)phenyl)-1,3,5-triazine (**TR1**), 2,4,6-tris(4-(3,6-di-tert-butyl-carbazol-9-yl)phenyl)-1,3,5-triazine (**TR2**) and 2,4,6-tris(4-((9-hexyl-carbazol-3-yl)ethynyl)phenyl)-1,3,5-triazine (**TR3**) are illustrated in Fig. 1, and the calculated IR spectra (except of **TR2**) are listed in Table S1 and Fig. S1. The ground state of these structures belongs to the *C*_3_ point group symmetry. As one can see in Table S1, the stationary points are characterized by the absence of imaginary frequencies, and are vibrationally stable.

To find the most appropriate functional for the absorption spectra calculation, we have performed a series of trials using hybrid functionals with different amounts of the exact HFE [34]. The results obtained are collected in Table 1. A strong correlation between the HFE (%) and the *S*_0_→*S*_1_ transition energy and intensity (oscillator strength) was found (Fig. S2). Except for the ωB97XD and CAM-B3LYP functionals, the following correlation coefficients were found: **TR1** and **TR2** (*R* ^2^ = 0.9817), **TR3** (*R* ^2^ = 0.9866) for transition energy; **TR1** and **TR2** (*R* ^2^ = 0.9995), **TR3** (*R* ^2^ = 0.9690) for oscillator strength. Note that the aforementioned correlations are presented for the hybrid functionals only. The range-separated functionals used in this study, namely, CAM-B3LYP and ωB97XD, were not involved in the correlation because they provide a different scheme for electron transition energy. As one can see in Table 1, the increased amount of HFE causes a rise in the transition energy. The range-separated functionals provide the same trend.

**Table 1:** Energy and intensity of the *S*_0_→*S*_1_ transition [in hexane, the polarizable continuum model (PCM) using integral equation formalism (IEFPCM) model] in the absorption spectra of the species **TR1**–**TR3** as functions of the Hartree-Fock exchange (HFE) contribution in the exchange-correlation functional

Functional	HFE (%)^a^	TR1			TR2			TR3		
		λ (nm)	*E* (eV)	*f*	λ (nm)	*E* (eV)	*f*	λ (nm)	*E* (eV)	*f*
Exp.^b^		389	3.19		397	3.12		394	3.14	
B3LYP-30	30	385	3.22	0.730	390	3.18	0.793	400	3.10	2.311
B3LYP	20	434	2.86	0.589	439	2.82	0.643	440	2.82	1.837
PBE0	25	410	3.03	0.655	415	2.99	0.713	419	2.96	2.065
mPW1PBE	25	410	3.03	0.655	415	2.99	0.713	419	2.96	2.067
CAM-B3LYP	19	327	3.79	1.151	331	3.75	1.234	352	3.52	2.936
BMK	42	355	3.49	0.898	360	3.45	0.971	374	3.32	2.673
ωB97XD	22	315	3.93	1.314	318	3.89	1.403	343	3.62	3.081
M062X	54	334	3.71	1.054	338	3.67	1.137	354	3.51	2.892

^a^For the range-separated functionals the values correspond to the short-range exchange

^b^Experimental data for *n*-hexane solution [6]

**Fig. 2:**
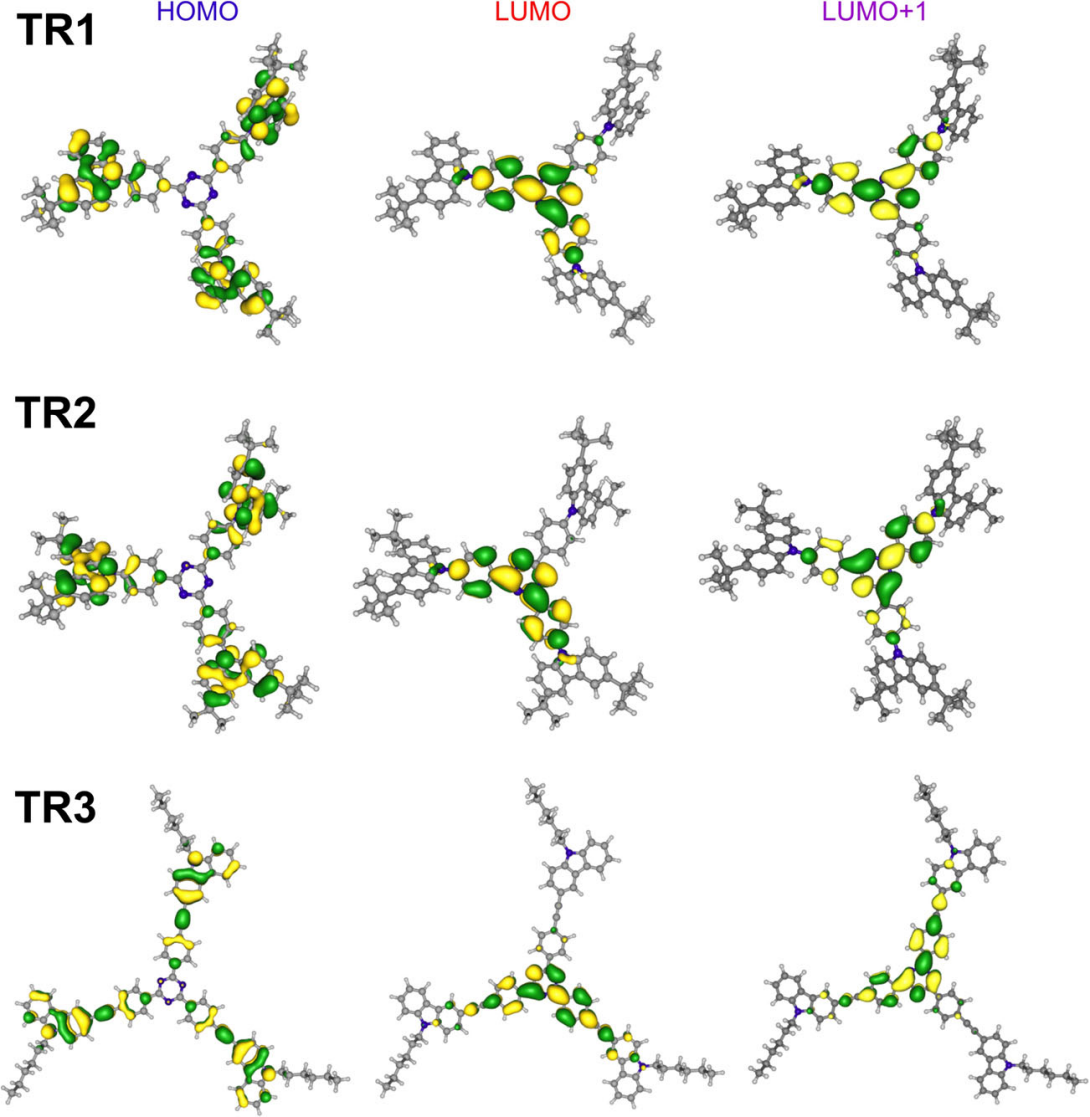
Frontier molecular orbitals—the highest occupied (HOMO) and the lowest unoccupied (LUMO)—of the studied dyes **TR1**–**TR3**

Herein, the custom-defined scheme (B3LYP-30) [18, 20, 21] provides the best fit with the experimental data (3.19 eV) [17], and overestimates the transition energy by only 0.032 eV (Table 1). The regular B3LYP functional gave a strong underestimation of the transition energy (about 0.330 eV), which is a known limitation of this functional for the CT states [34]. All other hybrid functionals demonstrate the same underestimation trend (Table 1). The use of range-separated functionals like CAM-B3LYP usually ensures more adequate energies for the CT states, but in our case this type of functional strongly overestimated the fluorescence-responsible *S*_1_ state energy (Table 1). This is due to the fact that the first S_1_ state for the **TR1**–**TR3** molecules combines the CT nature with local π→π*-excitation. As can be seen from Fig. 2, the HOMO and LUMO wave-functions are characterized by nonzero expansion coefficients in the same common part of the molecular space (phenyl ring nearest to the triazine core). This is a very important property of efficient light-harvesting end light-emissive materials. Such HOMO–LUMO “overlapping” provides a large electric dipole transition moment for light absorption and light emission processes (S_0_↔S_1_) [12], and, therefore, all the studied molecules are characterized by high values of oscillator strength for S_0_→S_1_ absorption as well as high fluorescence quantum yields for the S_1_→S_0_ emission channel (about 80% in non-polar media). For this reason, **TR1**–**TR3** compounds can be recommended not only as emitters for OLEDs [6, 35, 36] but also as light-harvesting materials for photovoltaic solar cells. Note that compounds **TR1**–**TR3** demonstrate high photoluminescence quantum yields reaching 0.85 [6].

### Nature of the absorption spectra

In this section, we discuss the calculated data on the absorption spectra of **TR1**–**TR3** molecules that are characterized by the close similar spectral properties (Table 2) due to the similar star-shaped structure (i.e., the same symmetry selection rules) and also the same D and A fragments for each of the three molecules. In Table 1, we selected only transitions with oscillator strengths >0.01. The first long-wavelength most intense absorption band for **TR1**–**TR3** molecules corresponds to the quasi-degenerate electronic transitions to the *S*_1_ and *S*_2_ states; the main configurations for both *S*_1_ and *S*_2_ states are the HOMO→LUMO+1 and HOMO→LUMO, respectively. Thus, here we account for the fact that LUMO and LUMO+1 are almost degenerate. The HOMO, LUMO and LUMO+1 wavefunctions for the **TR1**–**TR3** dyes are illustrated in Fig. 2, and the complete set of MOs involved in the electron transitions are presented in Figs. S3–S5.

**Table 2:** Calculated wave lengths (eV), oscillator strength (*f*) and orbital assignment for the quasi-degenerate S_0_→S_1_ and S_0_→S_2_ electronic transitions of **TR1**–**TR3** molecules by the B3LYP-30/6-31G(d,p) method within IEFPCM approach (*n*-hexane)

Transition	*E* (eV)	*f*	Assignment
TR1			
*S*_1_	3.22	0.730	HOMO→LUMO+1 (+48%) HOMO–1→LUMO (23%) HOMO–2→LUMO+1 (20%)
*S*_2_	3.22	0.731	HOMO→LUMO (+44%) HOMO–2→LUMO (+24%) HOMO–1→LUMO+1 (23%)
TR2			
*S*_1_	3.18	0.793	HOMO→LUMO+1 (+50%) HOMO–2→LUMO (+18%) HOMO–1→LUMO+1 (+17%)
*S*_2_	3.18	0.794	HOMO→LUMO (+45%) HOMO–1→LUMO (21%) HOMO–2→LUMO+1 (+19%)
TR3			
*S*_1_	3.10	2.311	HOMO→LUMO+1 (+40%) HOMO–1→LUMO (21%) HOMO–2→LUMO+1 (20%)
*S*_2_	3.10	2.311	HOMO→LUMO (+39%) HOMO–2→LUMO (+21%) HOMO–1→LUMO+1 (21%)

As one can see in Fig. 2, the HOMO orbital for **TR1**–**TR3** molecules is localized mainly on the carbazole moieties. Meanwhile, the LUMOs display electron densities primarily on the triazine core, and also on the phenyl bridges. Actually, both *S*_1_ and *S*_2_ states are characterized by their CT nature, but, at the same time, both HOMO and LUMO/LUMO+1 orbitals make large contributions to the common atoms of the linker (phenyl ring in the case of **TR1**, **TR2** molecules and phenyl-ethynyl fragment in **TR3**). This provides a large transition dipole moment for the *S*_0_–*S*_1_ and *S*_0_–*S*_2_ transitions. In fact, the larger the common area for the HOMO and LUMO/LUMO+1 wave-function, the higher the transition dipole moment and its oscillator strength. It can be seen from Table 2 that *S*_0_–*S*_1_ and *S*_0_–*S*_2_ transitions for the **TR1** and **TR2** molecules are characterized by almost the same oscillator strength values due to the same linker fragment. For the phenyl-ethynyl containing **TR3** compound, the intensity of the *S*_0_–*S*_1_ and *S*_0_–*S*_2_ transitions is three times higher than for the **TR1** and **TR2** compounds due to longer linker fragment in the **TR3** molecule. This fact is in good agreement with the experimental spectra regarding the first absorption band (*ε*_max_^0 − 0^ = 7 × 10^4^ M^− 1^cm^− 1^ for the **TR1** and **TR2** compounds, while for the **TR3** compound, *ε*_max_^0 − 0^ = 3.4 × 10^5^ M^− 1^cm^− 1^) [6].

**Fig. 3:**
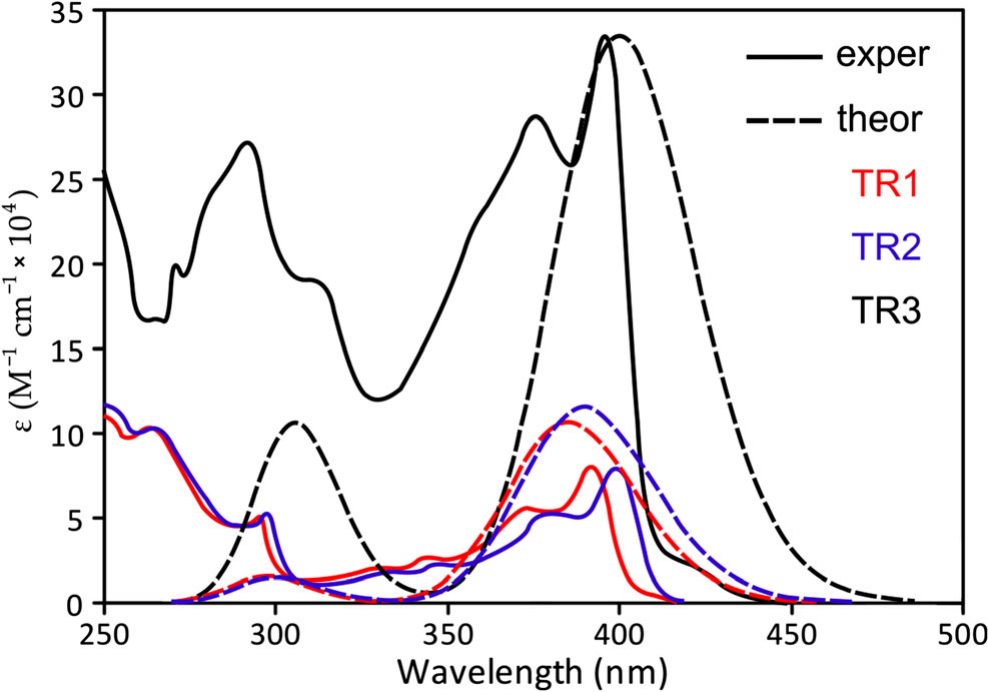
Plot of the calculated absorption spectra of the studied dyes **TR1**–**TR3**

We should stress that the experimental absorption spectrum of **TR1** (Fig. 3) exhibits three blue-shifted bands that are not reproduced by the vertical TD-DFT calculation. This is a progression of C–C vibrations (ν = 1276 cm^−1^), which correspond mostly to the triazine-phenyl link. Upon HOMO–LUMO excitation, this C–C bond becomes much stronger. The HOMO is a non-bonding orbital with respect to such a link, but the LUMO is a bonding orbital (Fig. 3); thus, the force constant of this mode is higher in the excited state. Such a strong change in the force field, and of the mode displacement upon excitation, leads to the occurrence of a long progression of the ν_C–C_ mode (1276 cm^−1^) in the first absorption band. The higher energy band in the absorption spectrum of **TR1** (at 4.16 eV), **TR2** (at 4.13 eV) and **TR3** (at 4.07 eV) corresponds to a local excitation of the ππ*-type in the carbazole moieties (Tables S3, S4).

### Solvatochromic effect

**Fig. 4:**
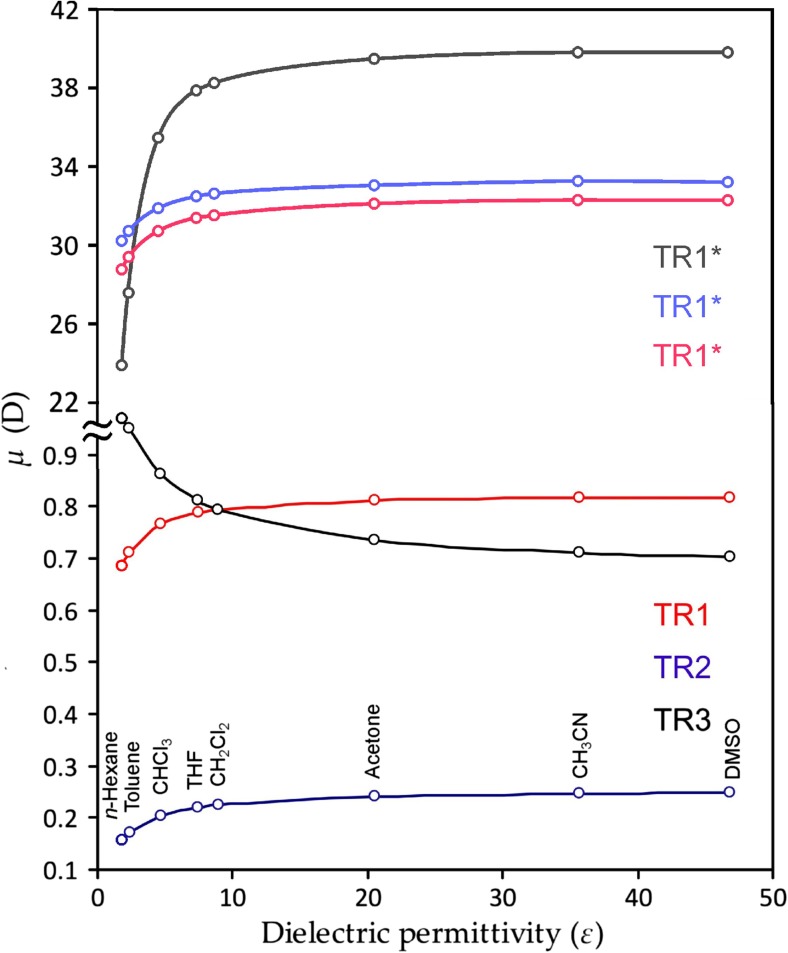
Dipole moment *μ* (D) for the ground (S_0_) and first excited (S_1_, *) states of the **TR1**–**TR3** dyes as a function of dielectric permittivity of solvent

An interesting experimental observation is that molecules **TR1**–**TR3** demonstrate a strong solvatochromic effect only in the fluorescence spectra, but not in the absorption spectra. The authors of a previous study [6] explained this fact by the strong differences in electronic structure of the ground and first singlet excited states for the studied **TR1**–**TR3** molecules. Therefore, we tracked the effect of solvent on molecular features such as permanent dipole moment of S_0_ and *S*_1_ electronic states, and also on the energy of *S*_1_ state, since the latter has a CT nature. The calculated data of the dipole moment dependence are presented in Fig. 4. Despite the symmetrical core of the molecular graph ©_3_ symmetry point group), the presence of carbazole ligands causes occurrence of a weak permanent dipole moment in the ground state of **TR1**–**TR3** molecules (Fig. 4).

**Fig. 5:**
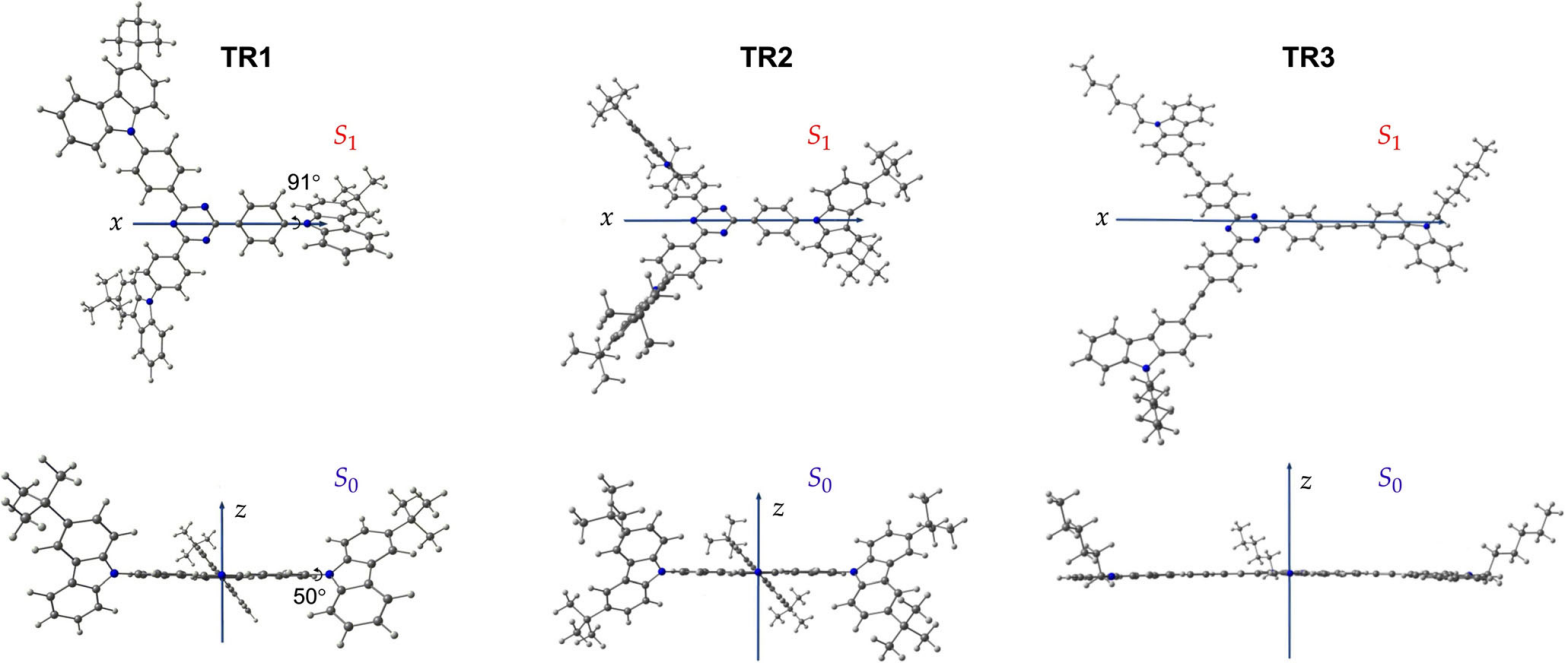
Orientation of the permanent dipole moment vector for the ground (*S*_0_) and first excited (*S*_1_) singlet state of molecules **TR1**–**TR3**

The corresponding dipole moment vector is oriented along the main molecular axis perpendicular to the triazine core plain (Fig. 5). As one can see in Fig. 4, the values of the ground state dipole moment (*μ*) are relatively small, and varying in the range of 0.1–1.0 D. This is because of the quasi-*C*_3_ symmetry, which almost excludes the existence of a permanent dipole moment for the **TR1**–**TR3** molecules in the ground state. As expected, the dipole moment values correlate with the dielectric permittivity of the solvents (Fig. 4). With a rise in solvent polarity, the *μ* value reaches a maximum in the case of **TR1** and **TR2**. At the same time, the corresponding *μ* values for **TR3** tend towards a minimum (Fig. 4). But, in all cases, the variation in the *μ* value is not more than 0.25 D between the non-polar cyclohexane solvent and the strongly polar acetonitrile. A similar correlation occurs for the *S*_0_–*S*_1_ vertical transition energies vs. dielectric permittivity. Both the theoretical and experimental energies decrease towards the rise in solvent polarity (Table 3). The calculated transition energies, however, are more robust to a change of solvent; in practice, this effect is much more pronounced [6].

**Table 3:** The vertical (vert.) and adiabatic (ad.) *S*_0_–*S*_1_ transition energies (eV) for the **TR1**–**TR3** molecules as a function of dielectric permittivity of solvent (theoretical values calculated using the B3LYP-30 scheme)

Dye	*n*-Hexane (*ε* = 1.88)		Toluene (*ε* = 2.37)		CHCl_3_ (*ε* = 4.71)		THF (*ε* = 7.43)		CH_2_Cl_2_ (*ε* = 8.93)		Acetone (*ε* = 20.49)		CH_3_CN (*ε* = 35.69)	
	Exp	Theor	Exp	Theor	Exp	Theor	Exp	Theor	Exp	Theor	Exp	Theor	Exp	Theor
TR1 (vert.)	3.12	3.22	3.22	3.18	3.27	3.19	3.28	3.2	3.3	3.2	3.33	3.2	3.35	3.2
TR1 (ad.)	3.19	2.17	2.86	2.01	2.58	1.64	2.55	1.49	2.48	1.44	2.38	1.29	2.26	1.24
TR2 (vert.)	3.12	3.18	3.15	3.18	3.2	3.19	3.24	3.2	3.24	3.2	3.26	3.2	3.35	3.2
TR2 (ad.)	3.05	2.05	2.79	1.9	2.53	1.55	2.52	1.4	2.42	1.36	2.32	1.22	2.13	1.17
TR3 (vert.)	3.15	3.1	3.15	3.05	3.18	3.06	3.17	3.05	3.19	3.05	3.23	3.05	3.24	3.05
TR3 (ad.)	3.1	2.79	2.74	2.68	2.54	2.37	2.45	2.23	2.4	2.18	2.23	2.06	2.13	2.02

The *μ* value and vector orientation change crucially upon electronic excitation of **TR1**–**TR3** molecules into the *S*_1_ state (Figs. 4, 5). We predicted an increase of >30 times the permanent dipole moment for the* S*_1_ exited state of molecules **TR1**–**TR3**, which is caused by the strong charge separation upon the* S*_0_–*S*_1_ electronic transition of CT nature.

A strong electric polarization of the **TR1**–**TR3** molecules in the *S*_1_ excited state provides strong stabilization of the excited molecule in polar solvents in comparison with nonpolar media. This means that more polar solvents provide stronger solvation of the excited-state molecule, which leads to a decrease in* S*_1_ state energy. This statement is in perfect qualitative agreement with experimental observations: the fluorescence wavelength increases strongly with the rise of solvent polarity. At the same time, the energy of the vertical* S*_0_→*S*_1_ transition (in absorption) is almost insensitive for the solvent effect, which means a strong increase in Stokes shift (Δν) with the rise in solvent polarity (experimentally, the Δν values change from the 615 cm^−1^ in nonpolar hexane to 9842 cm^−1^ in highly polar acetonitrile). Though our IEFPCM-B3LYP-30/6-31G(d) calculations strongly overestimate quantitatively the solvation energy of the* S*_1_ state of **TR1**–**TR3**, the overall tendency of the *S*_1_ energy vs. solvent polarity dependence is in good agreement with the experimental data.

It is interesting to note that the conformational structure of the excited state **TR1** and **TR2** molecules is almost the same as the ground state structure, except for the dihedral angle between the triazine core and one of the carbazole moiety (50° in *S*_0_ vs. 90° in the *S*_1_ state). Such symmetry distortion is a clear manifestation of the Jahn-Teller effect for the quasidegenerate *S*_1_ and *S*_2_ states, which should be strictly degenerate within the strict *C*_3_ symmetry point group constraints. For the **TR3** molecule, the excited state conformational structure is the same as the ground state structure

## Conclusions

In this work, we have presented a computational study of the absorption spectra for a series of star-shaped compounds containing both D (carbazole) and A (2,4,6-triphenyl-1,3,5-triazine) moieties bound through various linking bridges. These compounds demonstrate solvent-sensitive absorption in the whole visible range depending on solvent polarity. This is due to the strong charge-polarization of the studied molecules upon excitation into the *S*_1_ excited state of CT nature. It has been confirmed by the very high permanent dipole moment for the *S*_1_ excited state (>30 D) rather than for the *S*_0_ state (≤1 D). As a result, the solvatochromic effect should be observed only in fluorescence spectra, but not in absorption spectra, in complete agreement with experimental data.

An interesting feature of the *S*_0_-*S*_1_ transition for the studied compounds is a high intensity in the absorption spectrum, which is unusual for the CT transition. This result can be explained by the fact that the corresponding HOMO and LUMO+1 orbitals possess large contributions at the common atoms of the linker, which provides a large transition dipole moment for the *S*_0_-*S*_1_ transition. Actually, the solvent-dependent fluorescence for the studied compounds can be well explained by the localized CT excited state concept, which should be applicable to related D-A star-shaped systems.

The C_3_ symmetry point group for the studied systems determines that the* S*_1_ and* S*_2_ states are strictly degenerate in the vertical approximation. However, upon geometry relaxation in the excited state, these* S*_1_ and* S*_2_ states are split due to the Jahn-Teller effect. A study of this effect will be the subject of a future detailed investigation. Finally, we note that the studied star-shaped D–A compounds can be used to trigger color in OLEDs as controlled by the solvent.

## Electronic supplementary material

Below is the link to the electronic supplementary material.

ESM 1 (DOC 5987 kb)
