# Supplementary material for: Solvatochromic effect in absorption and emission spectra of star-shaped bipolar derivatives of 1,3,5-triazine and carbazole. A time-dependent density functional study
Source: J Mol Model. 2017 Feb 4;23(2):55. doi: 10.1007/s00894-017-3234-y (PMC5306056; doi:10.1007/s00894-017-3234-y)
Supplement: Supplementary file 1 — (DOC 5987 kb) [file 894_2017_3234_MOESM1_ESM.doc]

**Solvatochromic Effect in Absorption and Emission Spectra of Star-Shaped Bipolar Derivatives of 1,3,5-Triazine and Carbazole. A Time-Dependent Density Functional Study**

**Gleb V. Baryshnikov,^*,†,‡^ Sergey V. Bondarchuk,^‡^ Valentina A. Minaeva,^‡^ Hans Ågren^†^ and Boris F. Minaev^†,‡^**

*^†^Division of Theoretical Chemistry and Biology, School of Biotechnology, KTH Royal Institute of Technology, 10691 Stockholm, Sweden.*

*E-mail: glebar@theochem.kth.se*

*^‡^Department of Chemistry and Nanomaterials Science, Bogdan Khmelnitsky Cherkasy National University, blvd. Shevchenko 81, 18031 Cherkasy, Ukraine*

**SUPPORTING INFORMATION**

***Journal of Molecular Modeling***

**LIST OF SUPPLEMENTARY FIGURES AND TABLES:**

| **Table S1.** The calculated infrared spectra of the species **TR1** and **TR3**………………………………………………….. | 3 |
| --- | --- |
| **Figure S1.** Plots of the calculated IR spectra of the species **TR1** and **TR3** with the applied Lorentzian smearing……… | 11 |
| **Figure S2.** Correlation between the transition energies (left) or oscillator strengths (right) and the percentage of the Hartree-Fock exchange……………………………………………………………………………………………………. | 11 |
| **Figure S3.** Molecular orbitals which are involved into the most intense transitions in the UV-vis spectrum of **TR1**…… | 12 |
| **Figure S4.** Molecular orbitals which are involved into the most intense transitions in the UV-vis spectrum of **TR2**…… | 13 |
| **Figure S5.** Molecular orbitals which are involved into the most intense transitions in the UV-vis spectrum of **TR3**…… | 14 |

**Table S1. The calculated infrared spectra of the species TR1 and TR3**

| **Mode** | **TR1** | | **TR3** | |
| --- | --- | --- | --- | --- |
|  | ***v* (cm^–1^)** | ***I*, (km/mol)** | ***v* (cm^–1^)** | ***I*, (km/mol)** |
| 1 | 5.5 | 0.0028 | 1.2 | 0.0841 |
| 2 | 6.1 | 0.0202 | 1.8 | 0.0626 |
| 3 | 6.1 | 0.0202 | 2.9 | 0.0584 |
| 4 | 8.3 | 0.0001 | 4.3 | 0.1223 |
| 5 | 13.4 | 0.0115 | 4.8 | 0.0845 |
| 6 | 13.4 | 0.0115 | 5.0 | 0.0745 |
| 7 | 20.8 | 0.0106 | 10.0 | 0.0139 |
| 8 | 20.8 | 0.0106 | 10.2 | 0.0158 |
| 9 | 26.5 | 0.0098 | 10.7 | 0.0117 |
| 10 | 34.8 | 0.021 | 15.9 | 0.0144 |
| 11 | 34.8 | 0.0209 | 15.9 | 0.0138 |
| 12 | 40.4 | 0.0075 | 17.8 | 0.0004 |
| 13 | 49.7 | 0.1545 | 23.7 | 0.0057 |
| 14 | 49.7 | 0.1545 | 28.4 | 0.0061 |
| 15 | 51.8 | 0.0024 | 28.6 | 0.0065 |
| 16 | 64.9 | 4.9462 | 33.5 | 0.0025 |
| 17 | 64.9 | 4.9459 | 36.7 | 0.0767 |
| 18 | 65.4 | 0.06 | 36.8 | 0.0778 |
| 19 | 76.1 | 0.3161 | 38.1 | 0.0007 |
| 20 | 78.1 | 4.3512 | 38.2 | 0.0008 |
| 21 | 78.1 | 4.3513 | 42.9 | 0.2207 |
| 22 | 90.9 | 0.1467 | 46.1 | 0.0158 |
| 23 | 96.2 | 0.4079 | 46.7 | 0.0076 |
| 24 | 96.2 | 0.4081 | 46.8 | 0.0049 |
| 25 | 118.6 | 0.2067 | 59.6 | 0.0004 |
| 26 | 122.7 | 0.4989 | 59.6 | 0.0003 |
| 27 | 122.7 | 0.4988 | 63.0 | 0.0004 |
| 28 | 128.7 | 0.1916 | 67.1 | 0.0117 |
| 29 | 128.7 | 0.1917 | 67.5 | 0.0118 |
| 30 | 137.0 | 0.1892 | 77.9 | 0.0007 |
| 31 | 155.7 | 0.4366 | 88.1 | 0.4452 |
| 32 | 162.9 | 2.0189 | 95.9 | 0.0765 |
| 33 | 162.9 | 2.0186 | 100.7 | 0.0023 |
| 34 | 170.7 | 1.194 | 100.9 | 0.0024 |
| 35 | 170.7 | 1.1943 | 102.1 | 0.0022 |
| 36 | 171.5 | 0.1251 | 106.0 | 0.1418 |
| 37 | 183.2 | 0.1653 | 106.1 | 0.1434 |
| 38 | 184.0 | 0.1842 | 109.1 | 0.0610 |
| 39 | 184.0 | 0.1842 | 109.2 | 0.0600 |
| 40 | 234.9 | 0.1108 | 120.4 | 1.0609 |
| 41 | 234.9 | 0.1108 | 125.7 | 0.0555 |
| 42 | 243.5 | 0.0238 | 125.8 | 0.0554 |
| 43 | 248.7 | 0.3684 | 131.8 | 0.0063 |
| 44 | 248.7 | 0.3687 | 132.0 | 0.0014 |
| 45 | 248.8 | 0.152 | 132.2 | 0.0025 |
| 46 | 263.8 | 0.07 | 142.2 | 0.0711 |
| 47 | 263.8 | 0.07 | 142.7 | 0.0772 |
| 48 | 271.8 | 0.0643 | 144.9 | 0.0020 |
| 49 | 279.5 | 0.1079 | 155.5 | 0.7733 |
| 50 | 279.5 | 0.1329 | 155.6 | 0.7723 |
| 51 | 279.5 | 0.1329 | 159.3 | 1.3672 |
| 52 | 292.7 | 1.627 | 165.3 | 0.1221 |
| 53 | 292.7 | 1.627 | 165.3 | 0.1201 |
| 54 | 293.9 | 0.0476 | 174.2 | 0.0476 |
| 55 | 295.8 | 4.0524 | 186.1 | 0.0462 |
| 56 | 295.8 | 4.0522 | 186.1 | 0.0466 |
| 57 | 298.9 | 0.1108 | 202.3 | 0.0129 |
| 58 | 323.7 | 0.0375 | 217.3 | 0.4405 |
| **Mode** | **TR1** | | **TR3** | |
|  | ***v* (cm^–1^)** | ***I*, (km/mol)** | ***v* (cm^–1^)** | ***I*, (km/mol)** |
| 60 | 331.6 | 0.1225 | 225.4 | 0.1087 |
| 61 | 332.9 | 0.2005 | 243.8 | 0.3574 |
| 62 | 332.9 | 0.2002 | 243.9 | 0.3774 |
| 63 | 332.9 | 0.0254 | 249.0 | 0.0219 |
| 64 | 338.0 | 1.2126 | 249.2 | 0.0213 |
| 65 | 338.0 | 1.2128 | 249.3 | 0.0047 |
| 66 | 350.2 | 1.336 | 251.5 | 5.0824 |
| 67 | 350.2 | 1.3359 | 253.4 | 0.4575 |
| 68 | 356.7 | 0.2539 | 253.5 | 0.5496 |
| 69 | 364.0 | 0.7491 | 253.7 | 0.5749 |
| 70 | 373.5 | 1.1306 | 281.2 | 0.0040 |
| 71 | 373.5 | 1.1308 | 285.3 | 1.3445 |
| 72 | 404.4 | 0.5554 | 285.7 | 1.3766 |
| 73 | 412.2 | 6.9643 | 297.9 | 0.0644 |
| 74 | 412.2 | 6.9641 | 298.0 | 0.0674 |
| 75 | 417.6 | 0.0613 | 302.4 | 0.0095 |
| 76 | 423.1 | 0.0016 | 318.2 | 4.5625 |
| 77 | 423.8 | 23.1729 | 318.2 | 4.5440 |
| 78 | 423.8 | 23.1719 | 323.7 | 0.3539 |
| 79 | 435.5 | 0.0919 | 325.1 | 1.3429 |
| 80 | 435.5 | 0.092 | 325.1 | 1.2917 |
| 81 | 436.0 | 2.7813 | 332.3 | 1.0110 |
| 82 | 446.4 | 8.0463 | 378.4 | 0.1469 |
| 83 | 446.4 | 8.0451 | 378.4 | 0.1473 |
| 84 | 447.7 | 1.2802 | 397.7 | 0.9251 |
| 85 | 454.6 | 0.3767 | 414.0 | 15.7148 |
| 86 | 454.6 | 0.3768 | 414.0 | 15.7357 |
| 87 | 456.6 | 1.8309 | 414.7 | 0.0022 |
| 88 | 459.7 | 9.4461 | 416.0 | 0.0279 |
| 89 | 459.7 | 9.446 | 416.0 | 0.0292 |
| 90 | 463.8 | 0.2429 | 430.4 | 0.0390 |
| 91 | 475.7 | 11.319 | 430.4 | 0.0046 |
| 92 | 475.7 | 11.3187 | 431.2 | 8.5748 |
| 93 | 478.0 | 2.0645 | 435.6 | 0.4391 |
| 94 | 488.2 | 30.3529 | 436.1 | 0.9920 |
| 95 | 488.2 | 30.3557 | 436.4 | 1.1556 |
| 96 | 512.3 | 1.0737 | 446.9 | 3.2216 |
| 97 | 512.3 | 1.0733 | 446.9 | 3.1846 |
| 98 | 530.9 | 11.7986 | 448.6 | 0.5956 |
| 99 | 536.1 | 1.9991 | 449.2 | 0.9920 |
| 100 | 536.1 | 1.9991 | 453.0 | 0.4412 |
| 101 | 539.5 | 5.3032 | 453.1 | 0.5495 |
| 102 | 578.8 | 2.6806 | 453.9 | 2.1699 |
| 103 | 578.9 | 0.5939 | 457.4 | 14.6547 |
| 104 | 578.9 | 0.5938 | 457.4 | 14.5307 |
| 105 | 594.6 | 0.4604 | 476.4 | 0.3335 |
| 106 | 594.6 | 0.4603 | 481.1 | 1.2825 |
| 107 | 595.9 | 8.6098 | 481.1 | 1.2724 |
| 108 | 613.2 | 0.9216 | 489.3 | 0.2571 |
| 109 | 637.7 | 2.187 | 489.3 | 0.2256 |
| 110 | 637.7 | 2.1868 | 489.5 | 5.8515 |
| 111 | 639.4 | 5.0255 | 526.9 | 0.0224 |
| 112 | 644.2 | 2.9541 | 536.3 | 3.4695 |
| 113 | 644.2 | 2.9537 | 536.3 | 3.4686 |
| 114 | 644.8 | 0.1869 | 540.9 | 0.0179 |
| 115 | 650.0 | 3.7678 | 541.0 | 0.0172 |
| 116 | 650.0 | 3.7679 | 556.1 | 16.7361 |
| 117 | 650.7 | 20.1034 | 567.2 | 0.3733 |
| 118 | 662.8 | 1.8299 | 568.2 | 3.0356 |
| 119 | 662.8 | 1.8302 | 568.3 | 3.0358 |
| 120 | 667.8 | 4.2592 | 578.2 | 0.9133 |
| **Mode** | **TR1** | | **TR3** | |
|  | ***v* (cm^–1^)** | ***I*, (km/mol)** | ***v* (cm^–1^)** | ***I*, (km/mol)** |
| 122 | 687.1 | 4.7415 | 578.4 | 1.2078 |
| 123 | 701.1 | 2.0506 | 595.4 | 0.0564 |
| 124 | 701.1 | 2.0522 | 595.5 | 0.0576 |
| 125 | 706.3 | 0.0062 | 597.4 | 0.0017 |
| 126 | 738.6 | 9.0598 | 614.4 | 0.0214 |
| 127 | 738.6 | 9.0606 | 621.4 | 6.0197 |
| 128 | 744.2 | 11.8363 | 621.4 | 6.0470 |
| 129 | 746.1 | 3.7966 | 621.7 | 9.1713 |
| 130 | 746.1 | 3.7971 | 628.7 | 9.9331 |
| 131 | 746.4 | 0.4648 | 628.7 | 9.9492 |
| 132 | 751.9 | 0.0077 | 629.7 | 3.5367 |
| 133 | 760.4 | 29.3327 | 649.3 | 7.1239 |
| 134 | 760.4 | 29.3326 | 649.3 | 7.0968 |
| 135 | 760.7 | 42.795 | 649.6 | 0.1651 |
| 136 | 773.5 | 1.5624 | 658.1 | 1.0420 |
| 137 | 773.5 | 1.5626 | 658.1 | 1.0410 |
| 138 | 784.1 | 14.407 | 658.2 | 0.0188 |
| 139 | 784.1 | 14.4103 | 688.1 | 0.0268 |
| 140 | 784.1 | 9.7475 | 688.1 | 0.0264 |
| 141 | 799.0 | 3.2728 | 690.2 | 16.5621 |
| 142 | 799.0 | 3.2719 | 690.3 | 16.5664 |
| 143 | 800.9 | 0.0006 | 704.3 | 0.0081 |
| 144 | 829.2 | 38.8905 | 736.5 | 4.2141 |
| 145 | 829.4 | 16.6821 | 736.6 | 4.0800 |
| 146 | 829.4 | 16.6822 | 736.6 | 3.2357 |
| 147 | 841.1 | 58.8939 | 737.9 | 1.3791 |
| 148 | 856.0 | 0.1603 | 740.7 | 1.3171 |
| 149 | 856.7 | 0.9935 | 740.8 | 1.5467 |
| 150 | 856.7 | 0.9933 | 741.0 | 19.7966 |
| 151 | 859.8 | 29.9865 | 749.1 | 0.5591 |
| 152 | 859.8 | 29.9911 | 749.2 | 0.5344 |
| 153 | 869.6 | 1.9162 | 749.3 | 0.8617 |
| 154 | 869.6 | 1.9177 | 751.4 | 10.8518 |
| 155 | 870.1 | 6.8162 | 751.4 | 10.9080 |
| 156 | 870.5 | 7.219 | 751.6 | 6.1570 |
| 157 | 871.7 | 23.2425 | 760.2 | 10.8456 |
| 158 | 871.7 | 23.2434 | 760.2 | 0.3393 |
| 159 | 877.6 | 4.9819 | 760.3 | 91.9102 |
| 160 | 877.6 | 4.9799 | 770.0 | 0.0131 |
| 161 | 887.6 | 6.9819 | 770.1 | 0.0130 |
| 162 | 904.7 | 6.1439 | 780.6 | 1.6375 |
| 163 | 904.7 | 4.4945 | 780.6 | 2.9817 |
| 164 | 904.7 | 4.4918 | 780.8 | 3.2188 |
| 165 | 931.8 | 1.572 | 785.0 | 43.0820 |
| 166 | 931.8 | 1.402 | 785.0 | 43.0732 |
| 167 | 931.8 | 1.4016 | 796.4 | 4.0439 |
| 168 | 935.5 | 0.1025 | 796.5 | 5.7939 |
| 169 | 935.5 | 0.1025 | 796.6 | 9.2326 |
| 170 | 935.7 | 10.9896 | 803.3 | 0.0884 |
| 171 | 937.1 | 0.8733 | 818.3 | 46.3327 |
| 172 | 937.1 | 0.8726 | 818.3 | 3.5652 |
| 173 | 937.3 | 3.1303 | 818.8 | 22.8184 |
| 174 | 944.1 | 0.5757 | 837.4 | 131.2973 |
| 175 | 944.1 | 0.5759 | 853.8 | 0.0008 |
| 176 | 944.1 | 1.4773 | 855.7 | 0.0098 |
| 177 | 951.5 | 0.6006 | 855.7 | 0.0106 |
| 178 | 951.5 | 0.6 | 859.2 | 0.0131 |
| 179 | 951.6 | 0.1794 | 859.4 | 0.0100 |
| 180 | 957.7 | 7.0022 | 859.5 | 0.0135 |
| 181 | 957.7 | 7.0031 | 860.9 | 16.1725 |
| 182 | 958.2 | 1.2203 | 861.0 | 16.3935 |
| **Mode** | **TR1** | | **TR3** | |
|  | ***v* (cm^–1^)** | ***I*, (km/mol)** | ***v* (cm^–1^)** | ***I*, (km/mol)** |
| 184 | 964.2 | 0.1105 | 868.2 | 37.0425 |
| 185 | 964.2 | 0.0746 | 868.3 | 36.8045 |
| 186 | 974.5 | 0.4367 | 871.0 | 0.0327 |
| 187 | 974.5 | 0.4365 | 871.1 | 0.0331 |
| 188 | 974.5 | 0.0023 | 885.4 | 14.6103 |
| 189 | 990.9 | 0.0034 | 901.1 | 2.5603 |
| 190 | 991.2 | 1.008 | 901.2 | 4.8324 |
| 191 | 991.2 | 1.0073 | 901.3 | 4.7760 |
| 192 | 994.4 | 2.2612 | 903.4 | 7.3044 |
| 193 | 994.4 | 2.2592 | 903.4 | 8.1672 |
| 194 | 994.8 | 0.0312 | 903.5 | 11.3904 |
| 195 | 1012.0 | 0.017 | 906.0 | 0.0992 |
| 196 | 1033.5 | 0.0033 | 906.3 | 20.6176 |
| 197 | 1034.4 | 23.8478 | 906.3 | 20.8543 |
| 198 | 1034.4 | 23.8524 | 921.0 | 0.4248 |
| 199 | 1036.3 | 4.9395 | 921.0 | 0.4311 |
| 200 | 1036.3 | 4.9385 | 921.1 | 0.4269 |
| 201 | 1036.3 | 0.2495 | 931.6 | 0.6617 |
| 202 | 1048.7 | 0.9096 | 931.7 | 0.6767 |
| 203 | 1048.8 | 1.0984 | 931.7 | 0.6907 |
| 204 | 1048.8 | 1.0982 | 942.6 | 0.2632 |
| 205 | 1054.6 | 0.0864 | 942.6 | 0.3409 |
| 206 | 1054.6 | 0.0869 | 943.0 | 0.2967 |
| 207 | 1054.6 | 0.8085 | 972.2 | 0.0048 |
| 208 | 1055.9 | 3.8168 | 972.3 | 0.0045 |
| 209 | 1055.9 | 3.8161 | 972.3 | 0.0043 |
| 210 | 1055.9 | 13.7645 | 983.9 | 0.0001 |
| 211 | 1077.4 | 0.0009 | 986.0 | 0.0240 |
| 212 | 1117.1 | 31.9466 | 986.1 | 0.0240 |
| 213 | 1117.1 | 31.9499 | 991.5 | 0.0030 |
| 214 | 1117.5 | 1.4467 | 991.6 | 0.0028 |
| 215 | 1135.2 | 1.336 | 994.0 | 0.1581 |
| 216 | 1138.2 | 4.6785 | 1006.7 | 0.8294 |
| 217 | 1138.2 | 4.6777 | 1006.7 | 0.8366 |
| 218 | 1142.9 | 0.8843 | 1006.8 | 0.8296 |
| 219 | 1142.9 | 0.8845 | 1010.9 | 0.0000 |
| 220 | 1143.1 | 0.028 | 1012.6 | 1.7924 |
| 221 | 1167.0 | 10.3278 | 1012.9 | 1.8268 |
| 222 | 1167.0 | 10.3297 | 1013.0 | 1.7753 |
| 223 | 1167.6 | 19.0212 | 1032.3 | 0.0017 |
| 224 | 1176.9 | 112.0866 | 1033.3 | 17.4645 |
| 225 | 1176.9 | 112.0939 | 1033.4 | 17.5601 |
| 226 | 1183.9 | 15.1214 | 1034.2 | 0.3423 |
| 227 | 1183.9 | 15.119 | 1034.3 | 3.1356 |
| 228 | 1183.9 | 0.1669 | 1034.3 | 2.5018 |
| 229 | 1202.2 | 0.3262 | 1037.4 | 2.4299 |
| 230 | 1203.5 | 104.3683 | 1037.4 | 2.5981 |
| 231 | 1203.5 | 104.3839 | 1037.5 | 2.6313 |
| 232 | 1216.3 | 0.8558 | 1051.4 | 9.5150 |
| 233 | 1216.3 | 0.856 | 1051.5 | 6.1943 |
| 234 | 1217.4 | 1.3462 | 1051.6 | 7.2427 |
| 235 | 1231.7 | 2.5189 | 1053.9 | 0.3460 |
| 236 | 1231.7 | 2.5172 | 1054.2 | 0.3460 |
| 237 | 1231.7 | 6.8162 | 1054.4 | 0.3583 |
| 238 | 1232.2 | 0.0211 | 1070.0 | 0.3617 |
| 239 | 1234.9 | 2.6003 | 1070.4 | 0.3802 |
| 240 | 1234.9 | 2.6019 | 1070.8 | 0.3819 |
| 241 | 1235.0 | 1.917 | 1073.9 | 0.0857 |
| 242 | 1256.5 | 35.4339 | 1081.0 | 6.3504 |
| 243 | 1256.5 | 35.4238 | 1081.0 | 6.3081 |
| 244 | 1256.5 | 117.3654 | 1081.4 | 2.3377 |
| **Mode** | **TR1** | | **TR3** | |
|  | ***v* (cm^–1^)** | ***I*, (km/mol)** | ***v* (cm^–1^)** | ***I*, (km/mol)** |
| 246 | 1266.3 | 24.9132 | 1134.9 | 13.0063 |
| 247 | 1266.5 | 44.3804 | 1134.9 | 12.9255 |
| 248 | 1285.2 | 97.6248 | 1140.3 | 7.0974 |
| 249 | 1285.2 | 97.6131 | 1140.3 | 6.7983 |
| 250 | 1285.4 | 65.0202 | 1140.4 | 2.5835 |
| 251 | 1320.8 | 11.426 | 1147.3 | 92.8740 |
| 252 | 1320.9 | 4.1443 | 1147.4 | 95.1054 |
| 253 | 1320.9 | 4.1382 | 1148.9 | 0.3134 |
| 254 | 1330.2 | 67.278 | 1151.8 | 225.6738 |
| 255 | 1330.2 | 67.285 | 1151.8 | 222.7672 |
| 256 | 1332.5 | 8.9408 | 1154.2 | 0.0674 |
| 257 | 1335.1 | 25.8211 | 1169.7 | 48.8855 |
| 258 | 1335.1 | 25.8186 | 1169.8 | 46.8965 |
| 259 | 1342.4 | 17.6834 | 1170.2 | 1.6977 |
| 260 | 1348.9 | 10.3998 | 1176.4 | 120.2325 |
| 261 | 1348.9 | 10.3987 | 1176.5 | 120.7404 |
| 262 | 1352.4 | 18.5822 | 1181.4 | 4.5304 |
| 263 | 1375.0 | 392.7298 | 1181.5 | 12.5592 |
| 264 | 1375.0 | 392.7388 | 1181.6 | 12.6648 |
| 265 | 1376.4 | 3.3285 | 1187.3 | 1.5328 |
| 266 | 1386.8 | 176.7989 | 1187.4 | 25.7811 |
| 267 | 1386.8 | 176.7921 | 1187.4 | 25.1393 |
| 268 | 1387.7 | 6.8058 | 1207.4 | 0.0018 |
| 269 | 1396.4 | 198.7094 | 1208.2 | 105.0456 |
| 270 | 1396.4 | 198.7069 | 1208.3 | 104.8983 |
| 271 | 1396.9 | 0.0356 | 1229.7 | 45.3647 |
| 272 | 1403.1 | 946.3475 | 1229.7 | 44.1898 |
| 273 | 1403.1 | 946.3389 | 1229.8 | 19.5900 |
| 274 | 1410.2 | 7.2242 | 1232.6 | 0.0006 |
| 275 | 1410.2 | 7.2236 | 1250.5 | 33.8396 |
| 276 | 1410.2 | 16.5705 | 1250.5 | 21.9630 |
| 277 | 1411.6 | 0.1191 | 1250.6 | 12.8240 |
| 278 | 1411.6 | 9.3425 | 1260.2 | 5.4513 |
| 279 | 1411.6 | 9.3433 | 1260.5 | 5.6433 |
| 280 | 1442.8 | 3.14 | 1260.6 | 5.6433 |
| 281 | 1442.8 | 5.3394 | 1265.0 | 140.3686 |
| 282 | 1442.8 | 5.3402 | 1265.0 | 111.3032 |
| 283 | 1445.3 | 0.3748 | 1265.1 | 28.3217 |
| 284 | 1454.6 | 260.5998 | 1292.4 | 3.6446 |
| 285 | 1454.6 | 260.6404 | 1292.5 | 3.1773 |
| 286 | 1457.3 | 2.3814 | 1292.6 | 1.7082 |
| 287 | 1471.7 | 49.2044 | 1305.4 | 53.1535 |
| 288 | 1471.7 | 49.2112 | 1305.4 | 52.2450 |
| 289 | 1472.2 | 5.1072 | 1305.5 | 1.8394 |
| 290 | 1493.2 | 531.4182 | 1324.2 | 3.9436 |
| 291 | 1493.2 | 531.4036 | 1324.2 | 3.9907 |
| 292 | 1495.5 | 0.7412 | 1324.3 | 0.2412 |
| 293 | 1496.8 | 0.0805 | 1327.6 | 1.5850 |
| 294 | 1496.8 | 0.0807 | 1327.6 | 2.3677 |
| 295 | 1496.8 | 0.0357 | 1327.8 | 2.9995 |
| 296 | 1501.0 | 1.9891 | 1328.0 | 18.3473 |
| 297 | 1501.0 | 1.9907 | 1328.1 | 62.3235 |
| 298 | 1501.1 | 0.6107 | 1328.2 | 62.1633 |
| 299 | 1503.2 | 13.0459 | 1328.4 | 12.4332 |
| 300 | 1503.2 | 13.0453 | 1328.5 | 10.5096 |
| 301 | 1503.2 | 19.4758 | 1336.7 | 7.1819 |
| 302 | 1515.9 | 9.0435 | 1336.8 | 10.9114 |
| 303 | 1515.9 | 9.0462 | 1337.1 | 10.5228 |
| 304 | 1516.0 | 19.0015 | 1340.3 | 0.4775 |
| 305 | 1521.7 | 4.5632 | 1340.9 | 34.5159 |
| 306 | 1521.7 | 4.5616 | 1341.2 | 41.4386 |
| **Mode** | **TR1** | | **TR3** | |
|  | ***v* (cm^–1^)** | ***I*, (km/mol)** | ***v* (cm^–1^)** | ***I*, (km/mol)** |
| 308 | 1522.4 | 47.463 | 1344.2 | 16.8140 |
| 309 | 1522.4 | 47.4565 | 1344.4 | 13.5719 |
| 310 | 1522.7 | 25.0025 | 1344.5 | 8.6021 |
| 311 | 1527.8 | 113.8114 | 1359.3 | 8.6365 |
| 312 | 1527.8 | 113.8135 | 1359.3 | 26.5109 |
| 313 | 1528.4 | 45.0238 | 1359.3 | 24.0519 |
| 314 | 1535.4 | 37.6396 | 1380.3 | 74.3664 |
| 315 | 1535.4 | 37.6403 | 1380.4 | 78.8533 |
| 316 | 1535.5 | 36.5866 | 1380.5 | 11.8414 |
| 317 | 1555.4 | 949.6842 | 1385.6 | 117.5535 |
| 318 | 1555.4 | 949.679 | 1386.0 | 167.6500 |
| 319 | 1561.1 | 441.9064 | 1386.2 | 57.9223 |
| 320 | 1561.1 | 441.9507 | 1391.5 | 404.3689 |
| 321 | 1563.5 | 0.2599 | 1391.6 | 385.6130 |
| 322 | 1612.4 | 0.5266 | 1392.3 | 4.9635 |
| 323 | 1619.4 | 119.3739 | 1401.5 | 980.0852 |
| 324 | 1619.4 | 119.3749 | 1401.5 | 981.3195 |
| 325 | 1626.6 | 2.7223 | 1415.2 | 11.0551 |
| 326 | 1628.1 | 57.2061 | 1415.8 | 12.0656 |
| 327 | 1628.1 | 57.2074 | 1415.9 | 11.2858 |
| 328 | 1631.9 | 0.3776 | 1417.7 | 22.0302 |
| 329 | 1633.6 | 69.4728 | 1417.7 | 17.9872 |
| 330 | 1633.6 | 69.4606 | 1417.9 | 17.6765 |
| 331 | 1654.0 | 21.1068 | 1421.2 | 1.1758 |
| 332 | 1654.0 | 42.6024 | 1421.4 | 37.3898 |
| 333 | 1654.0 | 42.5989 | 1421.5 | 37.6397 |
| 334 | 1659.6 | 217.1356 | 1428.9 | 1.3636 |
| 335 | 1659.6 | 217.1381 | 1429.0 | 1.3392 |
| 336 | 1659.8 | 1.5853 | 1429.0 | 1.3467 |
| 337 | 1676.6 | 0.1709 | 1443.0 | 0.0450 |
| 338 | 1676.6 | 0.1709 | 1447.8 | 212.6601 |
| 339 | 1676.7 | 0.4885 | 1447.8 | 212.8383 |
| 340 | 3036.7 | 23.4936 | 1450.7 | 0.0091 |
| 341 | 3036.7 | 23.4822 | 1476.6 | 8.3478 |
| 342 | 3036.7 | 34.6214 | 1476.6 | 8.3809 |
| 343 | 3037.9 | 37.8795 | 1476.8 | 0.4989 |
| 344 | 3037.9 | 37.8848 | 1498.5 | 14.5507 |
| 345 | 3037.9 | 0.019 | 1498.6 | 9.3762 |
| 346 | 3045.0 | 36.172 | 1498.7 | 4.8629 |
| 347 | 3045.0 | 36.1726 | 1499.7 | 20.3084 |
| 348 | 3045.1 | 42.9849 | 1499.8 | 16.9651 |
| 349 | 3106.2 | 2.0607 | 1499.8 | 5.3896 |
| 350 | 3106.2 | 2.0597 | 1501.4 | 38.8667 |
| 351 | 3106.2 | 14.605 | 1501.4 | 37.2764 |
| 352 | 3107.5 | 6.1514 | 1501.5 | 12.9855 |
| 353 | 3107.5 | 6.1489 | 1507.5 | 75.3549 |
| 354 | 3107.5 | 7.8222 | 1507.6 | 72.2413 |
| 355 | 3113.4 | 14.7384 | 1507.7 | 7.6923 |
| 356 | 3113.4 | 25.7607 | 1512.5 | 27.0786 |
| 357 | 3113.4 | 25.7455 | 1512.6 | 24.5525 |
| 358 | 3115.1 | 145.2957 | 1512.7 | 14.3425 |
| 359 | 3115.1 | 145.2847 | 1514.3 | 5.3812 |
| 360 | 3115.1 | 84.7832 | 1514.3 | 5.5558 |
| 361 | 3118.9 | 2.9011 | 1514.3 | 5.5353 |
| 362 | 3118.9 | 36.4034 | 1516.5 | 236.2434 |
| 363 | 3118.9 | 36.4034 | 1516.5 | 236.0673 |
| 364 | 3122.9 | 10.1357 | 1517.3 | 1.5427 |
| 365 | 3122.9 | 10.1425 | 1521.5 | 0.9210 |
| 366 | 3122.9 | 84.8906 | 1521.5 | 1.0637 |
| 367 | 3181.9 | 0.436 | 1521.6 | 0.9802 |
| 368 | 3181.9 | 0.4359 | 1528.0 | 4.9250 |
| **Mode** | **TR1** | | **TR3** | |
|  | ***v* (cm^–1^)** | ***I*, (km/mol)** | ***v* (cm^–1^)** | ***I*, (km/mol)** |
| 370 | 3188.0 | 17.6678 | 1528.0 | 5.9356 |
| 371 | 3188.0 | 17.6744 | 1530.8 | 281.9444 |
| 372 | 3188.1 | 1.1884 | 1530.8 | 281.4124 |
| 373 | 3191.0 | 19.1444 | 1531.2 | 1.5415 |
| 374 | 3191.0 | 19.1526 | 1547.7 | 1910.5150 |
| 375 | 3191.1 | 10.9002 | 1547.7 | 1910.5510 |
| 376 | 3205.3 | 57.5464 | 1551.8 | 135.8104 |
| 377 | 3205.3 | 57.5597 | 1551.8 | 134.9662 |
| 378 | 3205.4 | 20.6304 | 1557.6 | 0.0018 |
| 379 | 3206.5 | 14.6362 | 1594.1 | 0.0002 |
| 380 | 3206.5 | 14.6213 | 1608.3 | 90.8944 |
| 381 | 3206.5 | 0.8545 | 1608.3 | 96.2403 |
| 382 | 3211.1 | 9.6697 | 1608.7 | 0.5599 |
| 383 | 3211.1 | 9.6781 | 1609.8 | 436.4937 |
| 384 | 3211.2 | 4.4875 | 1609.8 | 441.8335 |
| 385 | 3213.2 | 2.7468 | 1629.6 | 1.1738 |
| 386 | 3213.2 | 2.7507 | 1629.6 | 1.6771 |
| 387 | 3213.2 | 1.8987 | 1629.6 | 1.7438 |
| 388 | 3218.4 | 6.6429 | 1647.1 | 19.0054 |
| 389 | 3218.4 | 6.6497 | 1647.1 | 308.1144 |
| 390 | 3218.4 | 15.0228 | 1647.1 | 289.9433 |
| 391 | 3223.8 | 7.8171 | 1660.1 | 0.5451 |
| 392 | 3223.8 | 7.8109 | 1660.3 | 128.8739 |
| 393 | 3223.8 | 12.2931 | 1660.3 | 129.1674 |
| 394 | 3232.4 | 0.0004 | 1675.3 | 34.7191 |
| 395 | 3232.9 | 1.2308 | 1675.3 | 23.5873 |
| 396 | 3232.9 | 1.2305 | 1675.3 | 13.6706 |
| 397 | 3233.9 | 5.6687 | 2303.1 | 528.9450 |
| 398 | 3233.9 | 5.6637 | 2303.1 | 528.9551 |
| 399 | 3234.4 | 0.0038 | 2303.7 | 0.0209 |
| 400 |  |  | 3008.9 | 4.9779 |
| 401 |  |  | 3009.1 | 4.7817 |
| 402 |  |  | 3009.1 | 5.2900 |
| 403 |  |  | 3014.4 | 5.4850 |
| 404 |  |  | 3014.5 | 5.2193 |
| 405 |  |  | 3014.6 | 5.3964 |
| 406 |  |  | 3024.9 | 56.5243 |
| 407 |  |  | 3025.0 | 55.1414 |
| 408 |  |  | 3025.0 | 61.2119 |
| 409 |  |  | 3034.5 | 1.3081 |
| 410 |  |  | 3034.6 | 1.3255 |
| 411 |  |  | 3034.7 | 1.3176 |
| 412 |  |  | 3037.9 | 14.0033 |
| 413 |  |  | 3037.9 | 14.4058 |
| 414 |  |  | 3038.0 | 14.9135 |
| 415 |  |  | 3038.9 | 34.3434 |
| 416 |  |  | 3038.9 | 37.8581 |
| 417 |  |  | 3039.0 | 40.5205 |
| 418 |  |  | 3046.1 | 0.7436 |
| 419 |  |  | 3046.3 | 0.7186 |
| 420 |  |  | 3046.3 | 0.7215 |
| 421 |  |  | 3054.4 | 71.5859 |
| 422 |  |  | 3054.4 | 72.4726 |
| 423 |  |  | 3054.5 | 72.3024 |
| 424 |  |  | 3066.2 | 27.7918 |
| 425 |  |  | 3066.3 | 27.4530 |
| 426 |  |  | 3066.3 | 28.1465 |
| 427 |  |  | 3077.9 | 24.6134 |
| 428 |  |  | 3078.0 | 24.5531 |
| 429 |  |  | 3078.0 | 24.6105 |
| 430 |  |  | 3101.9 | 29.0000 |
| **Mode** | **TR1** | | **TR3** | |
|  | ***v* (cm^–1^)** | ***I*, (km/mol)** | ***v* (cm^–1^)** | ***I*, (km/mol)** |
| 432 |  |  | 3102.0 | 27.6957 |
| 433 |  |  | 3106.5 | 62.2987 |
| 434 |  |  | 3106.5 | 62.3196 |
| 435 |  |  | 3106.6 | 64.6214 |
| 436 |  |  | 3112.4 | 46.3615 |
| 437 |  |  | 3112.4 | 54.5862 |
| 438 |  |  | 3112.4 | 31.5029 |
| 439 |  |  | 3181.0 | 0.5490 |
| 440 |  |  | 3181.0 | 0.5428 |
| 441 |  |  | 3181.0 | 0.5194 |
| 442 |  |  | 3188.5 | 5.4870 |
| 443 |  |  | 3188.6 | 5.2658 |
| 444 |  |  | 3188.6 | 5.4380 |
| 445 |  |  | 3195.5 | 14.5197 |
| 446 |  |  | 3195.5 | 11.6605 |
| 447 |  |  | 3195.6 | 12.2048 |
| 448 |  |  | 3200.0 | 27.7667 |
| 449 |  |  | 3200.0 | 28.4842 |
| 450 |  |  | 3200.1 | 28.1665 |
| 451 |  |  | 3203.4 | 9.8900 |
| 452 |  |  | 3203.4 | 10.3188 |
| 453 |  |  | 3203.4 | 0.1850 |
| 454 |  |  | 3203.8 | 12.9115 |
| 455 |  |  | 3203.8 | 14.9763 |
| 456 |  |  | 3203.9 | 0.8394 |
| 457 |  |  | 3204.1 | 7.2523 |
| 458 |  |  | 3204.1 | 7.2217 |
| 459 |  |  | 3204.2 | 7.1444 |
| 460 |  |  | 3208.9 | 40.6064 |
| 461 |  |  | 3208.9 | 34.7241 |
| 462 |  |  | 3209.0 | 30.2851 |
| 463 |  |  | 3213.3 | 13.3996 |
| 464 |  |  | 3213.3 | 8.9849 |
| 465 |  |  | 3213.4 | 10.5329 |
| 466 |  |  | 3229.4 | 0.0158 |
| 467 |  |  | 3229.9 | 4.6801 |
| 468 |  |  | 3230.0 | 4.5042 |
| 469 |  |  | 3230.9 | 2.5564 |
| 470 |  |  | 3230.9 | 2.3658 |
| 471 |  |  | 3231.5 | 0.0032 |


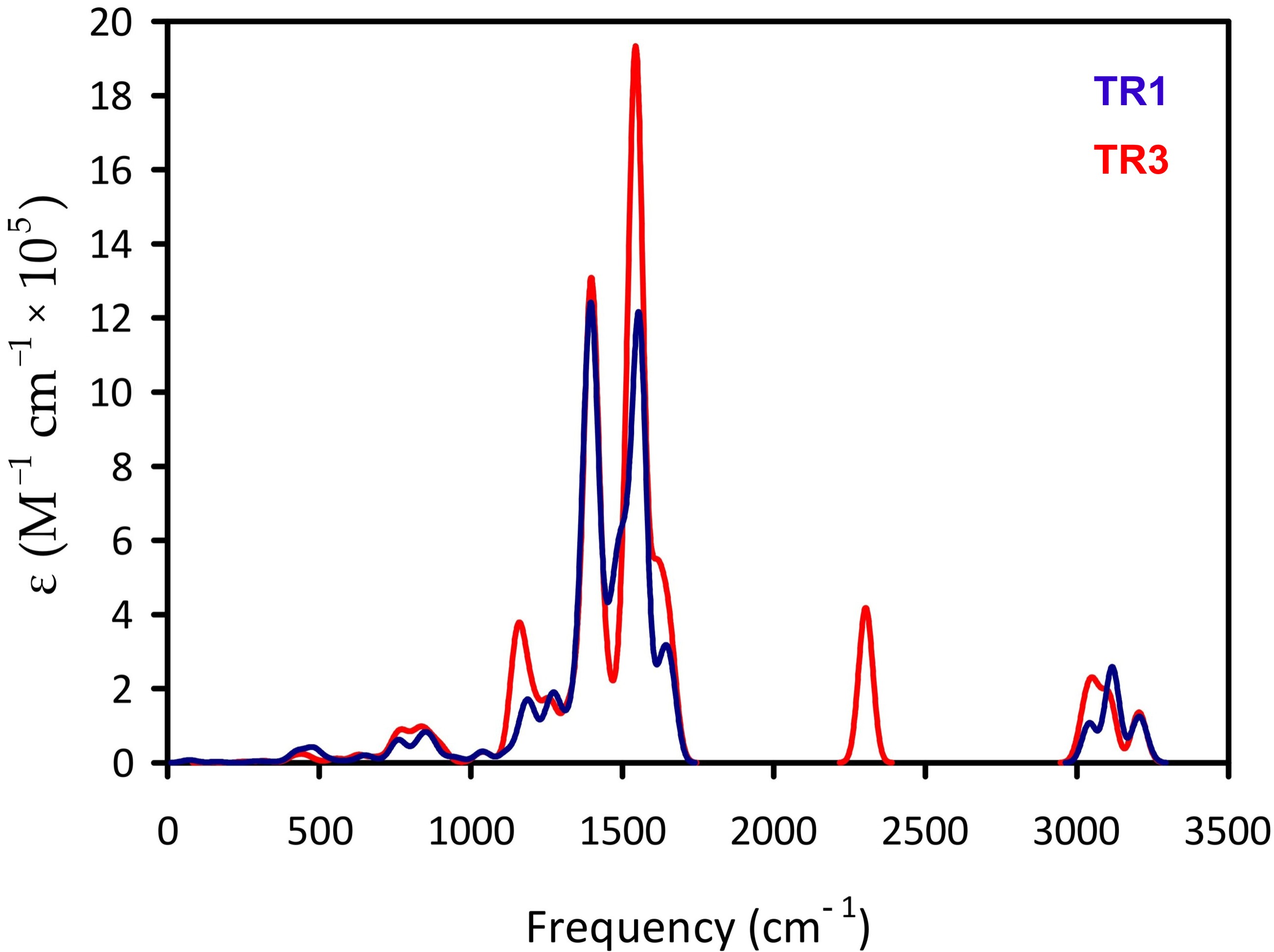


**Figure S1.** Plots of the calculated IR spectra of the species **TR1** and **TR3** with the applied Lorentzian smearing


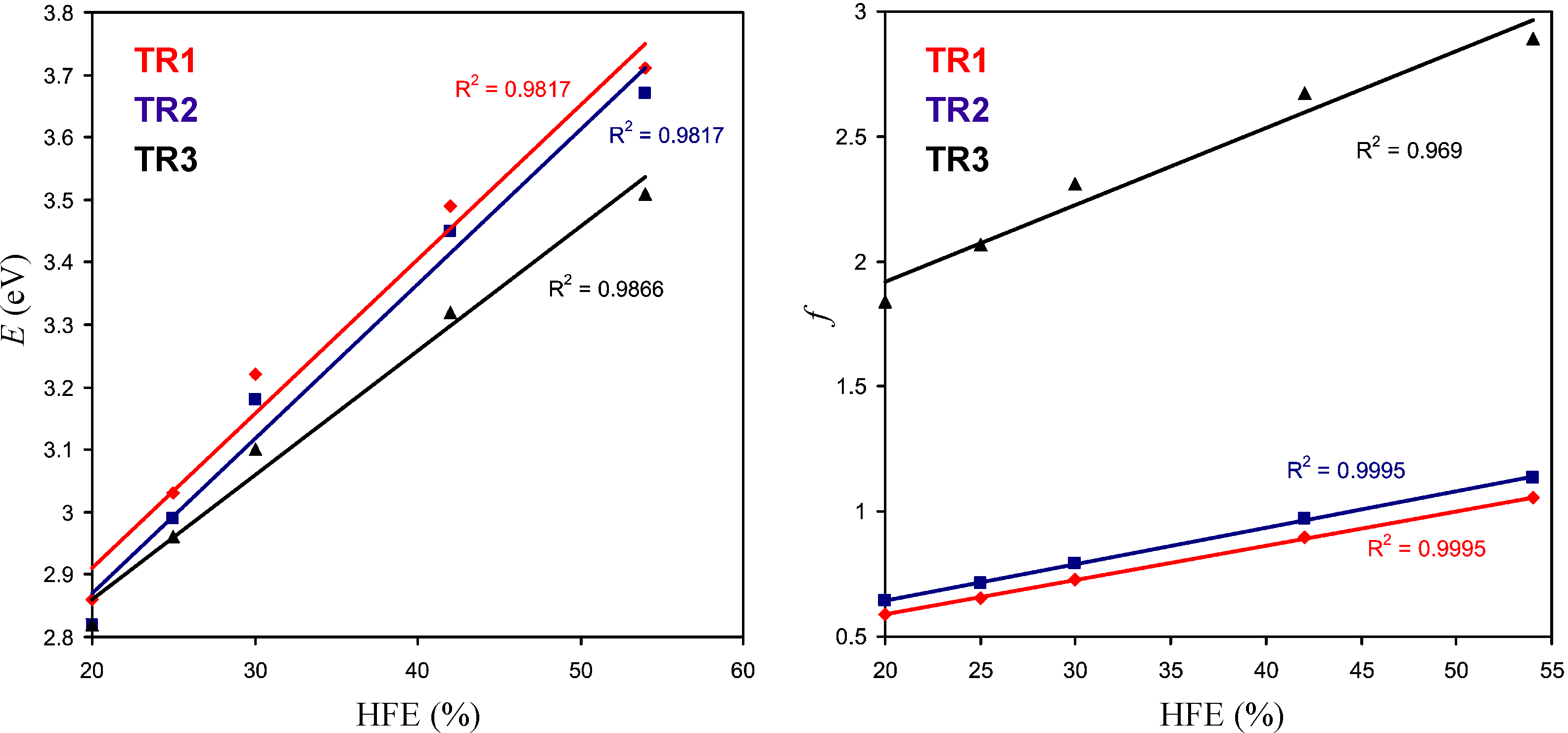


**Figure S2.** Correlation between the transition energies (left) or oscillator strengths (right) and the percentage of the Hartree-Fock exchange.


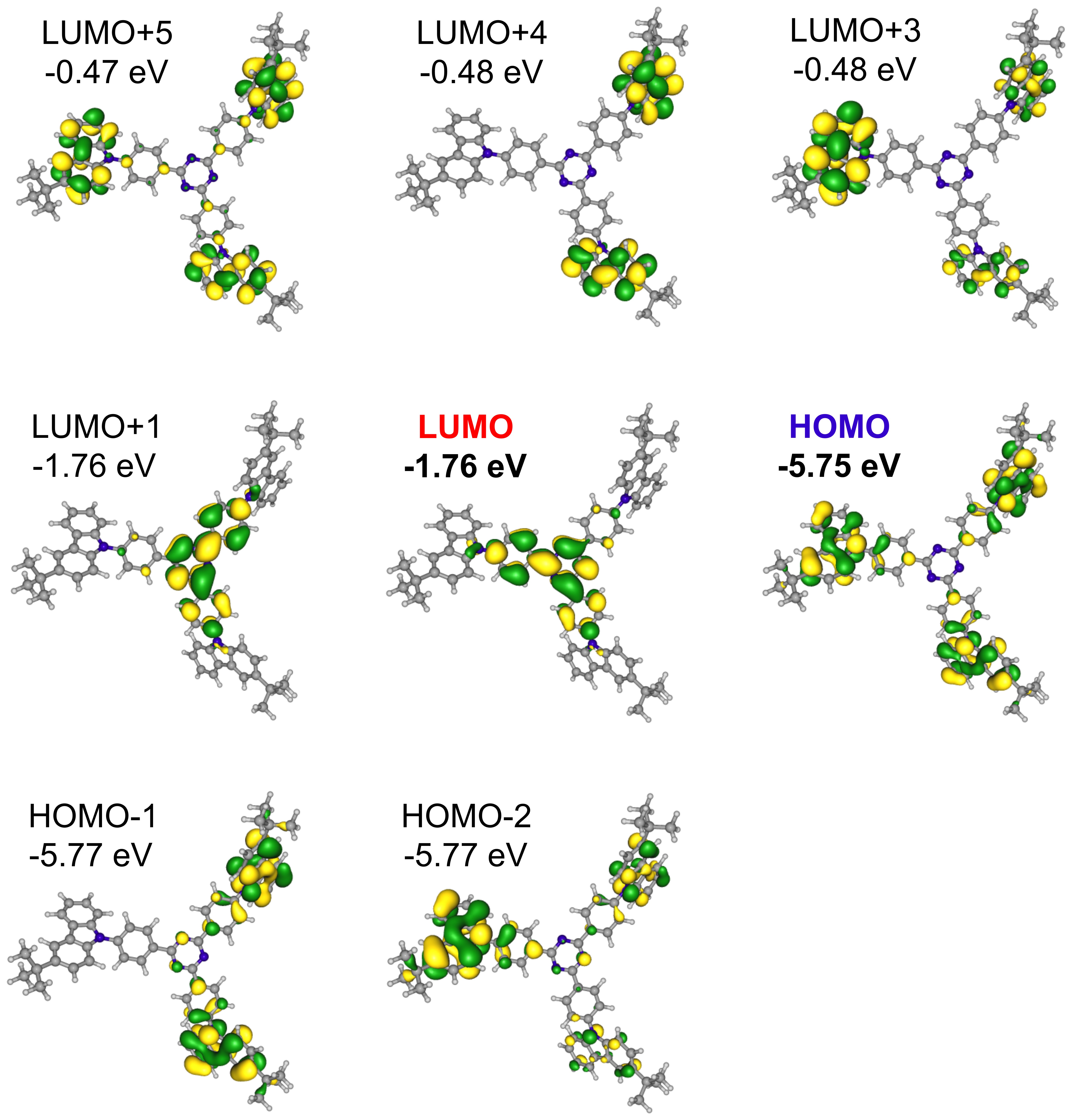


**Figure S3.** Molecular orbitals which are involved into the most intense transitions in the UV-vis spectrum of **TR1**


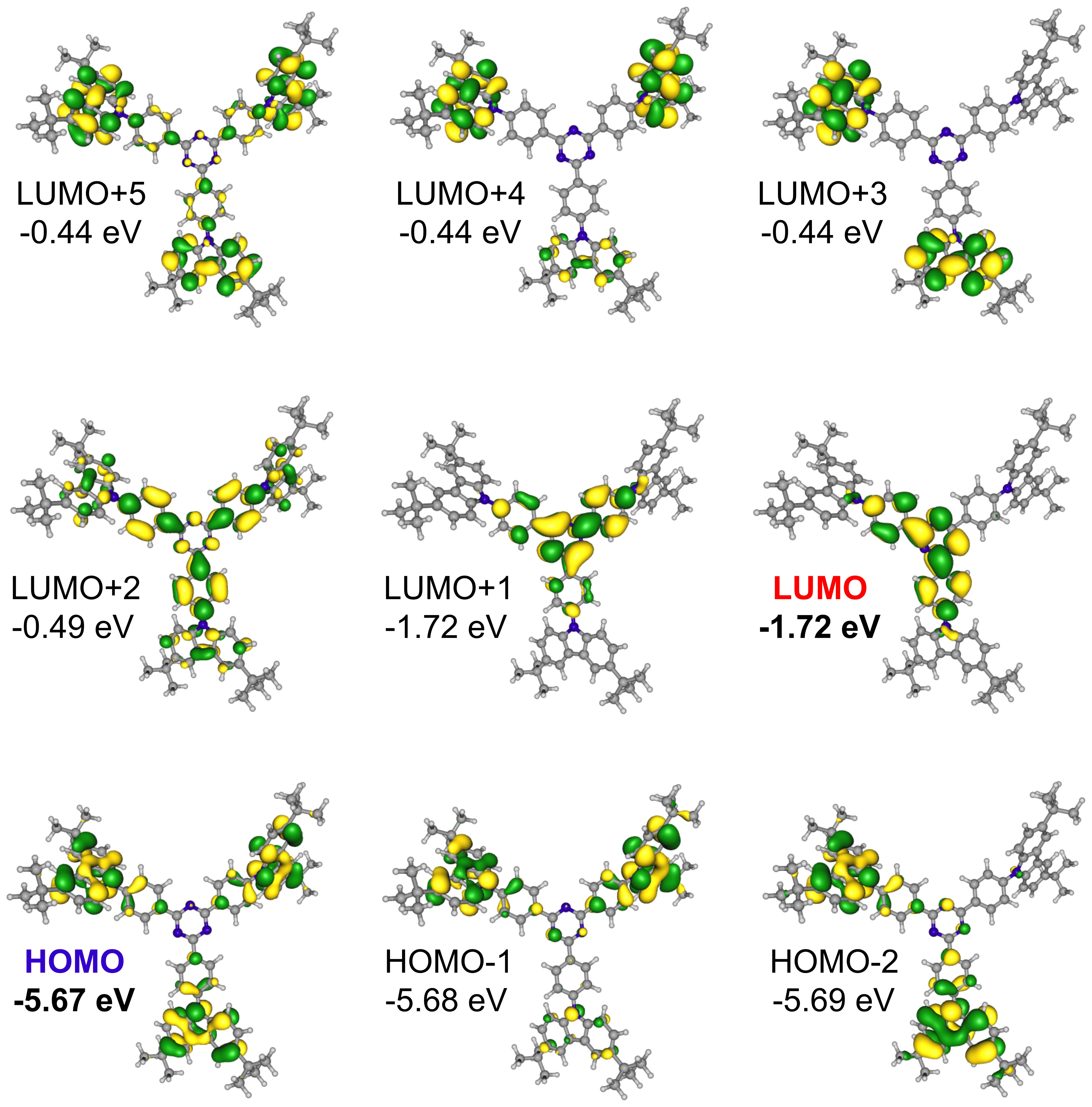


**Figure S4.** Molecular orbitals which are involved into the most intense transitions in the UV-vis spectrum of **TR2**


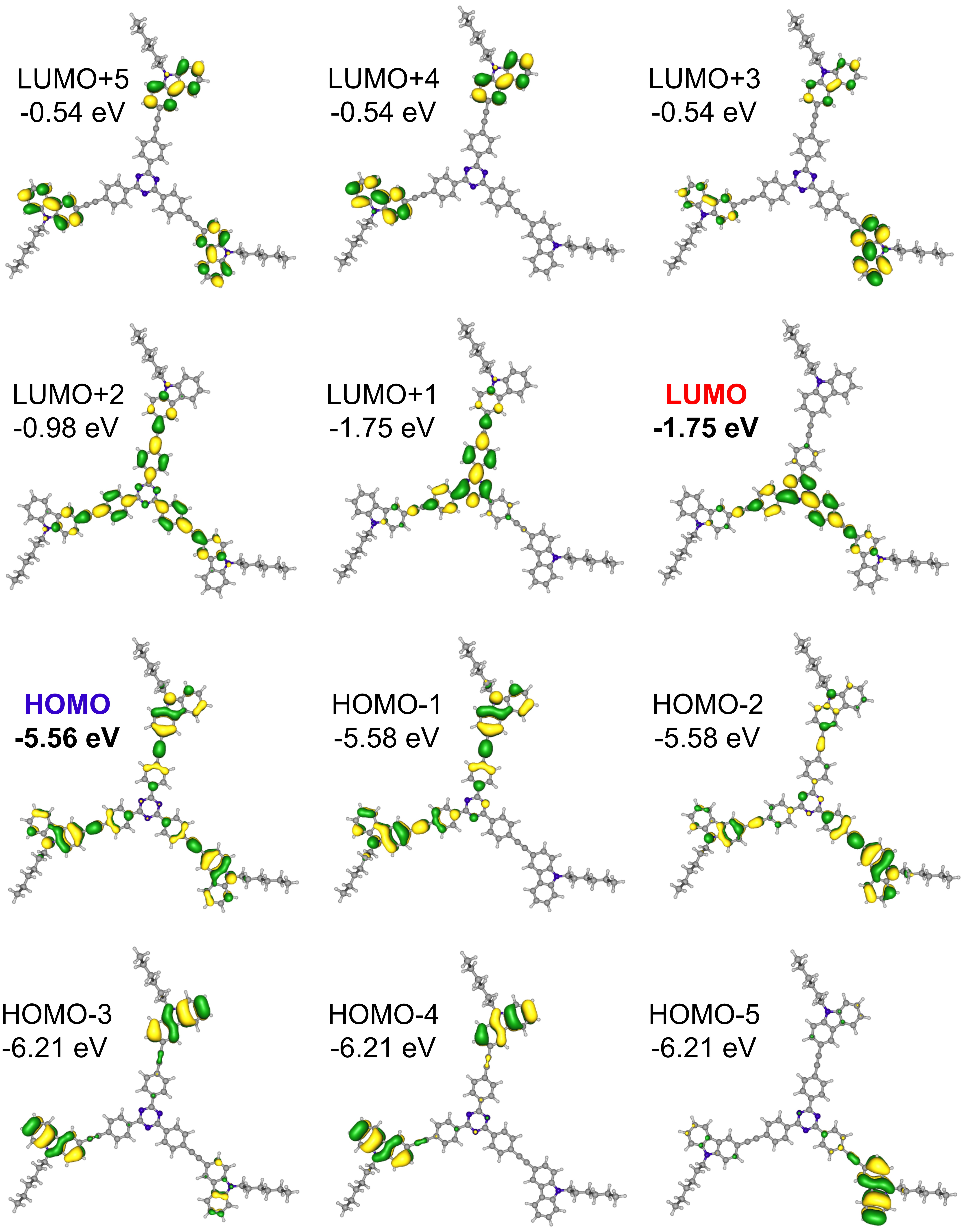


**Figure S5.** Molecular orbitals which are involved into the most intense transitions in the UV-vis spectrum of **TR3**
